# Role of the vacuolar ATPase in the *Alphavirus* replication cycle

**DOI:** 10.1016/j.heliyon.2018.e00701

**Published:** 2018-07-25

**Authors:** Ryan M. Schuchman, Ricardo Vancini, Amanda Piper, Denitra Breuer, Mariana Ribeiro, Davis Ferreira, Joseph Magliocca, Veronica Emmerich, Raquel Hernandez, Dennis T. Brown

**Affiliations:** a Department of Molecular and Structural Biochemistry, North Carolina State University, Raleigh, NC, USA; b Institute of Microbiology, Federal University of Rio de Janeiro, Rio de Janeiro, RJ, Brazil

**Keywords:** Virology, Cell biology

## Abstract

We have shown that *Alphaviruses* can enter cells by direct penetration at the plasma membrane (R. Vancini, G. Wang, D. Ferreira, R. Hernandez, and D. Brown, J Virol, 87:4352–4359, 2013). Direct penetration removes the requirement for receptor-mediated endocytosis exposure to low pH and membrane fusion in the process of RNA entry. Endosomal pH as well as the pH of the cell cytoplasm is maintained by the activity of the vacuolar ATPase (V-ATPase). Bafilomycin is a specific inhibitor of V-ATPase. To characterize the roll of the V-ATPase in viral replication we generated a Bafilomycin A1(BAF) resistant mutant of Sindbis virus (BRSV). BRSV produced mature virus and virus RNA in greater amounts than parent virus in BAF-treated cells. Sequence analysis revealed mutations in the E2 glycoprotein, T15I/Y18H, were responsible for the phenotype. These results show that a functional V-ATPase is required for efficient virus RNA synthesis and virus maturation in *Alphavirus* infection.

## 1 Introduction

**Fig. 1:**
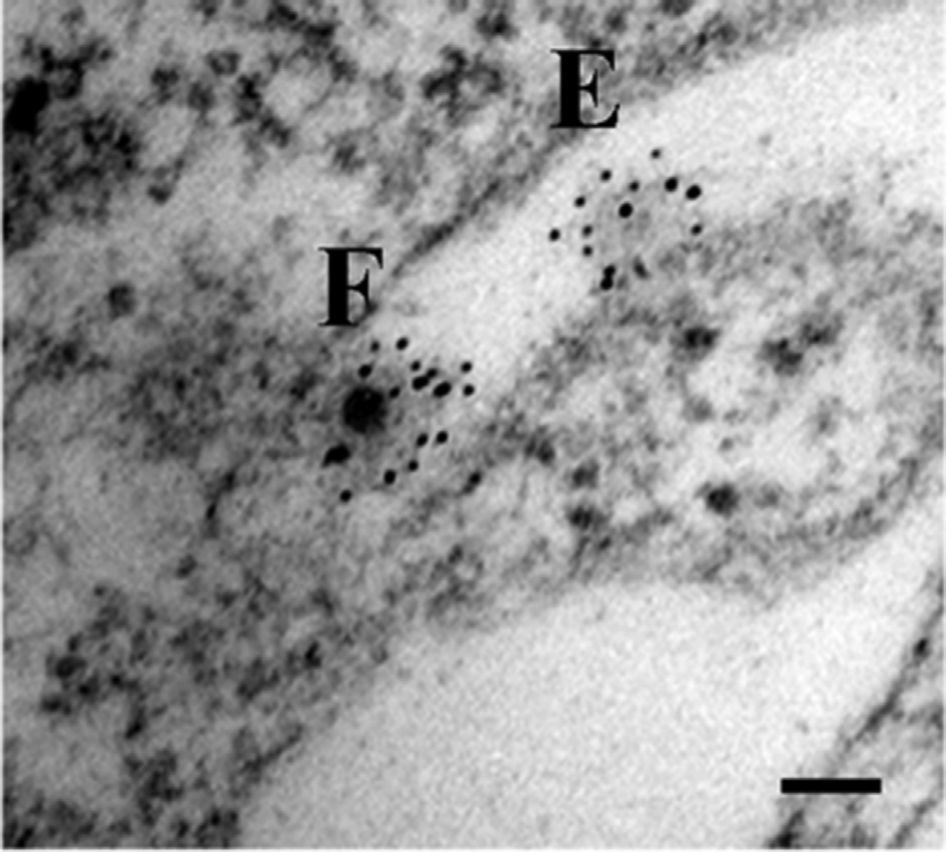
Electron micrograph of Sindbis virus entry events on a BHK cell surface. Cells were infected with SVHR at an MOI of 1000 at room temperature for 15 min; virus particles were treated with anti-Sindbis structural protein polyclonal antibodies, then with immunogold-labeled secondary antibody and processed for electron microscopy. “F” indicates a full virus particle; “E” indicates an empty particle.

Sindbis virus is the prototype *Alphavirus* in the *Togaviridae* family. These viruses consist of a positive-polarity, single-stranded RNA that is housed in an internally situated nucleocapsid that is in turn surrounded by an outer protein shell. A membrane, whose lipid composition is host-dependent, lies between the nucleocapsid and outer protein shell [1, 2]. The outer protein envelope is composed of trimers of the E1 and E2 protein heterodimers [1, 2]. The proteins of the outer shell provide the tools by which the virus attaches to the cell surface (E2) and a mechanism that facilitates the entry of the viral genome into the cell (E1) [3]. We have previously shown that this entry event takes place at the cell surface by direct penetration in a time and temperature dependent process with an activation energy (E_a_) of *ca.* 27 Kcal/mol [4]. We have also demonstrated that viral entry does not require endocytosis, acidification, or membrane fusion, as suggested by others [5, 6, 7, 8]. Past studies may have incorrectly classified entry as the pH-sensitive step due to the use of indirect reporters that addressed events downstream of entry, *i.e.* transcription or translation [9, 10]. The model suggesting that receptor-mediated endocytosis is the mechanism for virus entry was challenged by the development of an electron microscopy protocol that *directly* visualized the entry process [5, 6]. This protocol uses immunogold-labeling to facilitate the identification of *empty* virus particles on the surface of the cell that would have otherwise been overlooked after entry took place (Fig. 1).

Once the genome has entered the host cell, the viral polycistronic RNA is translated into 4 non-structural proteins, nsP1-4. nsP1 functions as a methyltransferase to add a methylguanosine cap to newly synthesized viral RNA [11, 12, 13, 14]. nsP2 is the protease that cleaves the nonstructural polypeptide and also is the viral helicase [15, 16, 17, 18, 19]. nsP3 is an accessory in the synthesis of the negative RNA strand [20, 21]; finally, nsP4 is the RNA-dependent RNA polymerase [22, 23, 24]. The structural proteins are translated from a subgenomic viral 26S RNA.

While Sindbis virus is an *Alphavirus*, it belongs to a broader group of arthropod-borne viruses, the arboviruses. The arboviruses are mainly represented in nature by three families (*Flaviviridae*, *Togaviridae, and Nairoviridae [formerly Bunyaviridae]*) and representative species include chikungunya virus, Mayaro virus, and (alphavirus), West Nile virus, dengue virus, yellow fever virus, Zika virus, (flavivirus), and Crimean-Congo Hemorrhagic Fever virus (*Nairoviridae*) [25]. A vaccine exists for yellow fever virus, but effective treatments or prophylactics for the others are still not available [26]. By fully characterizing the replication cycle of these viruses, using Sindbis virus as the accepted model system for *Alphaviruses*, new therapeutic targets and strategies may be identified and developed.

SVHR (Sindbis virus heat-resistant) is a strain of Sindbis virus that is structurally stable and has a particle-to-PFU ratio that approaches unity when properly prepared [27]. This characteristic ensures that nearly every virus particle is infectious making a morphological study of entry possible.

We have investigated the role that V-ATPase plays in the Sindbis virus replication cycle by producing a virus mutant of SVHR, resistant to the effects of bafilomycin A1 (BAF). BAF is a specific inhibitor of the Vacuolar ATPase (V-ATPase), known to be involved in controlling the pH of the organelles in the secretory pathway [28, 29]. Other known functions of the V-ATPase range from control of pH homeostasis to vesicular trafficking within the cell, whose functions are dependent upon an isoform of the V-ATPase. For instance, the a2 isoform is responsible for early endosome acidification, and the a3 isoform targets the Trans Golgi in the secretory pathway, other isoforms and combinations exist [30, 31, 32]. It has been shown previously that interruption of the secretory pathway by BAF results in an accumulation of Golgi-derived vesicles [33].

The goal of this study is to identify what the effect of an inhibited V-ATPase has on alphavirus replication by examining entry, RNA production, virus maturation, and egress of SVHR compared to a BAF-resistant Sindbis virus mutant (BRSV).

## 2 Results

### 2.1 Effect of metabolic inhibitors on *Alphavirus* entry

**Fig. 2:**
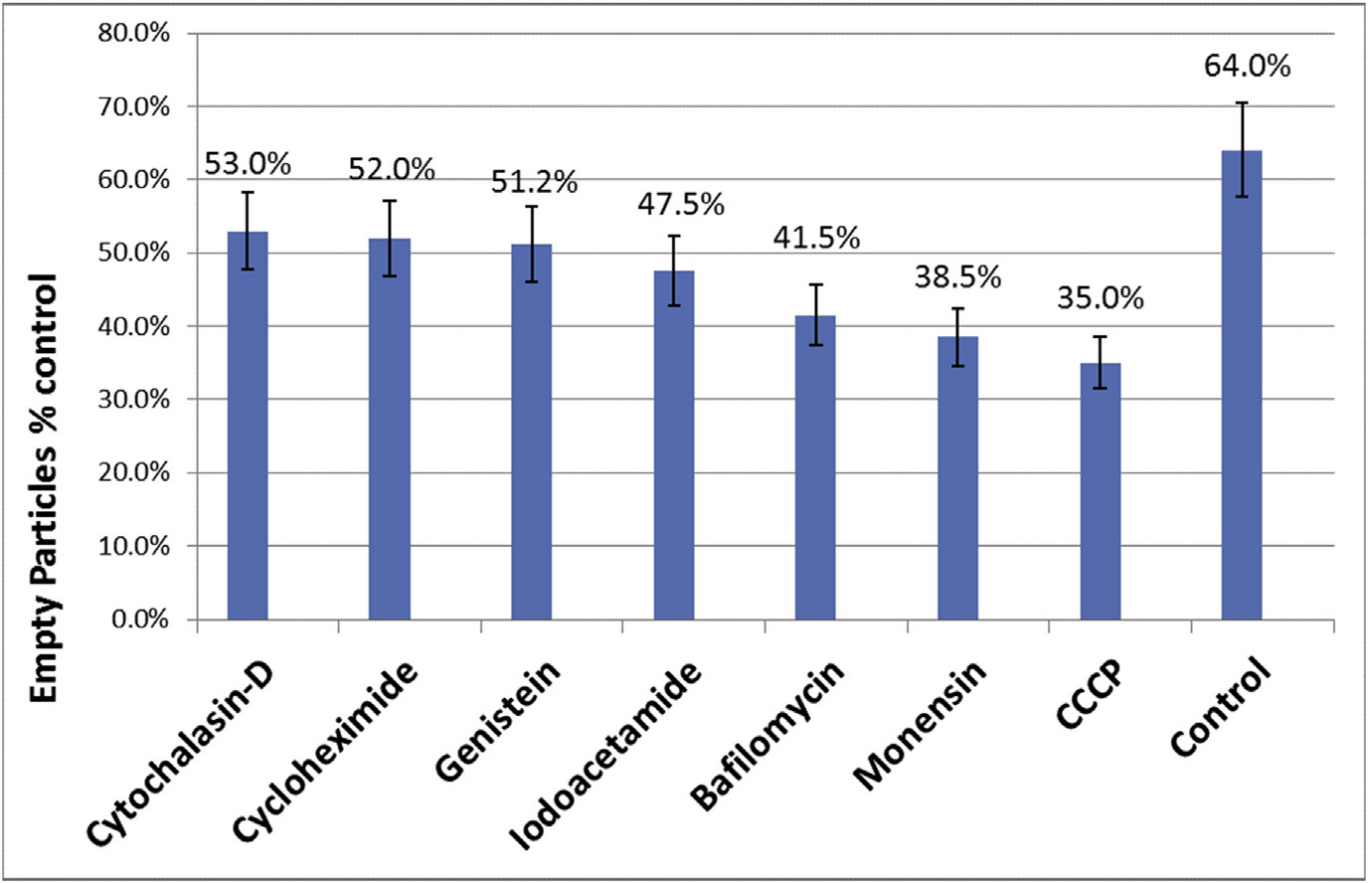
The Effect of Metabolic Inhibitors on *Alphavirus* Entry. BHK cells were untreated (control), treated with cytochalasin-D (50μM), cyclohexamide (100μM), genestin (100μM), CCCP (40μM), monensin (50 nM), or bafilomycin A1 (500 nM) for 1 hour. After treatment, cells were infected with SVHR for 15 min at room temperature, fixed, processed, and treated with primary, secondary antibodies, and counted as described in the text. Error bars account for a 10% error and the percent inhibition for each treatment is shown.

The effect of metabolic inhibitors on the entry of SVHR in Baby Hamster Kidney cells (BHK) was examined using the indicated concentration of each inhibitor shown in Fig. 2. After cells were treated for an hour with the respective inhibitor, the cells were infected at a multiplicity of 1,000 PFU/cell for 15 minutes at ambient temperature, fixed, processed, incubated with a primary anti-SVHR polyclonal antibody, and then incubated with immunogold beads-conjugated with secondary antibody. This high MOI is not for synchronization, but rather needed to ensure the observation of a sufficient number of virus particles on the surface of the cell. For each treatment, the number of full and empty particles out of a total of 100 was determined. In all cases, particles less than 60% of the full-sized diameter were not included in the dataset to ensure that the core of the virus particle would be in the plane of the thin section. That the appearance of empty particles is not an artifact of thin sectioning for the electron microscope is underscored by the fact that the appearance of empty particles is time and temperature dependent and follows Arrhenius kinetics. If control values are set as 100% and all the treatments are set as % of control Cytochalasin-D, cyclohexamide, and genistein were found to reduce the number of empty particles to approximately 80% of control value. Carbonyl cyanide-*m*-chlorophenylhydrazone (CCCP), monensin, and BAF were found to reduce the number of empty particles to approximately 55%, 60% and 65%, of control level respectively. The most significant inhibition of entry was seen in treatments with ionophores (CCCP and monensin). This indicates a requirement for membrane potential. A lesser effect was seen with BAF treatment, which also has a secondary effect on membrane potential. Endocytosis of virions or the presence of virus in endosomes was not seen in this study as was the case in our previous studies involving direct observation [4, 6].

### 2.2 Generation of a BAF-resistant virus

Twenty-two serial passages of SVHR in BHK cells treated with 100 nM BAF produced a mutant that was resistant to BAF [34]. The mutant virus was deemed BAF-resistant after the titer rose above 10^8^ PFU/mL, non-adapted virus grown in the presence of BAF produced a titer of 4 × 10^6^ PFU/mL. When the resulting mutant was sequenced, we discovered three mutations in the structural proteins: one in E1 (L222F) and two in E2 (T15I, Y18H). There were no mutations found in the replication (nonstructural) proteins.

### 2.3 Effect of BAF on alphavirus RNA replication

**Fig. 3:**
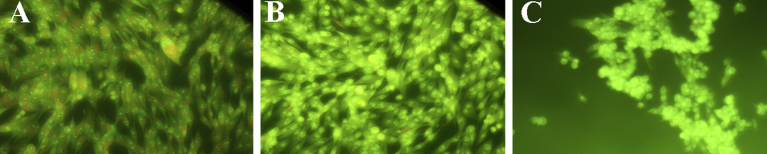
The Effect of BAf on endosome acidification. Monolayers of BHK cells were either untreated (A), treated with 100 nM BAF for 1 hour (B), or for 10 hours (C) before incubation with acridine orange and subsequent imaging. Cells with orange vesicles show acidic compartments (A) and cells lacking orange vesicles show endosome pH neutralization (B and C).

**Fig. 4:**
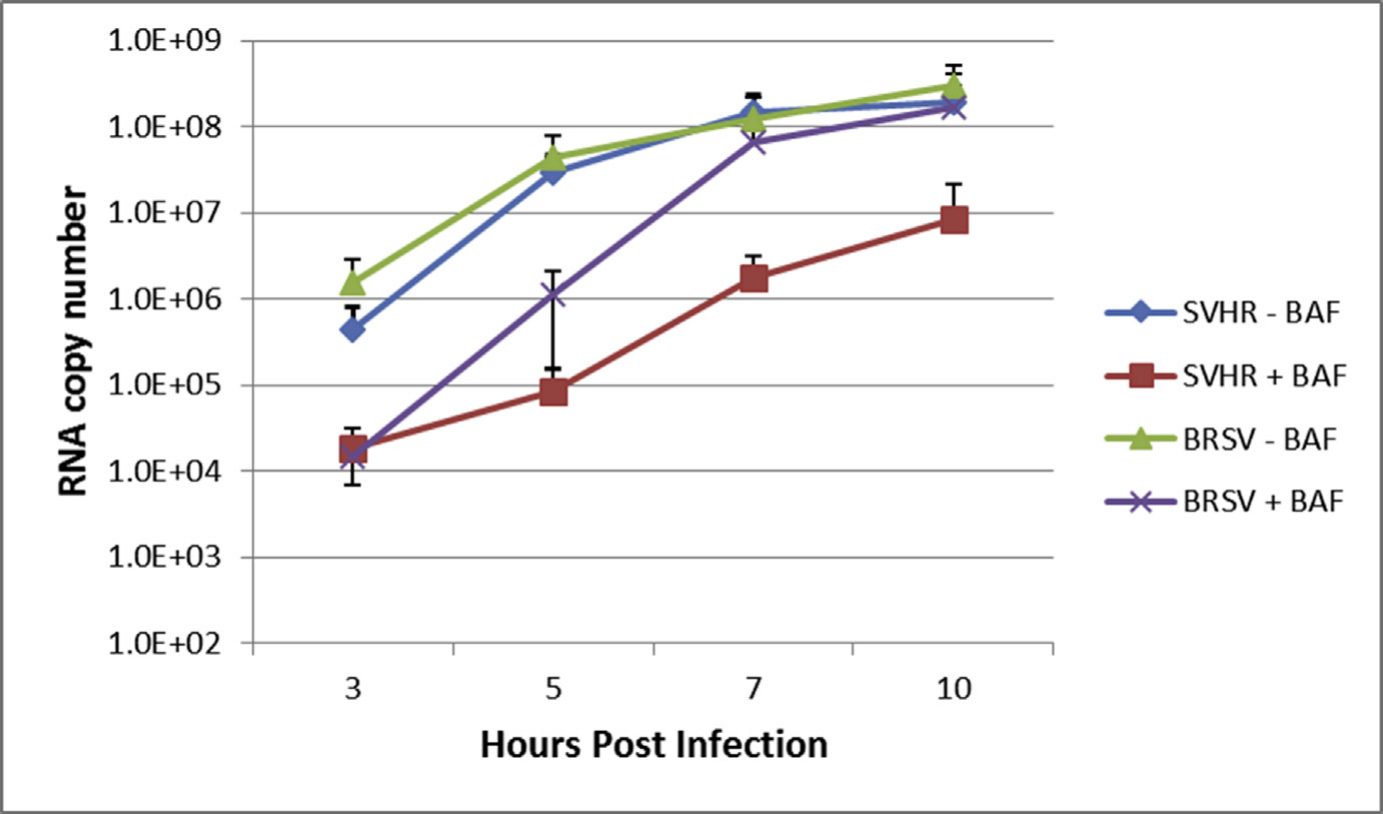
The effect of BAF on SVHR RNA production. BHK cells were pretreated with 100 nM BAF or tissue culture media at ambient temperature for 1 hour. Following this, cells were infected with the indicated virus (MOI = 10 PFU/cell) in media with 100 nM BAF or without BAF at ambient temperature. Cells were then incubated at 37 °C for the indicated amount of time in the presence or absence of BAF. RT-qPCR treatment groups are as follows: blue, SVHR without BAF; red, SVHR + 100 nM BAF; green, BRSV without BAF; purple, BRSV + 100 nM BAF. Experiment is an average of three biological replicates.

**Fig. 5:**
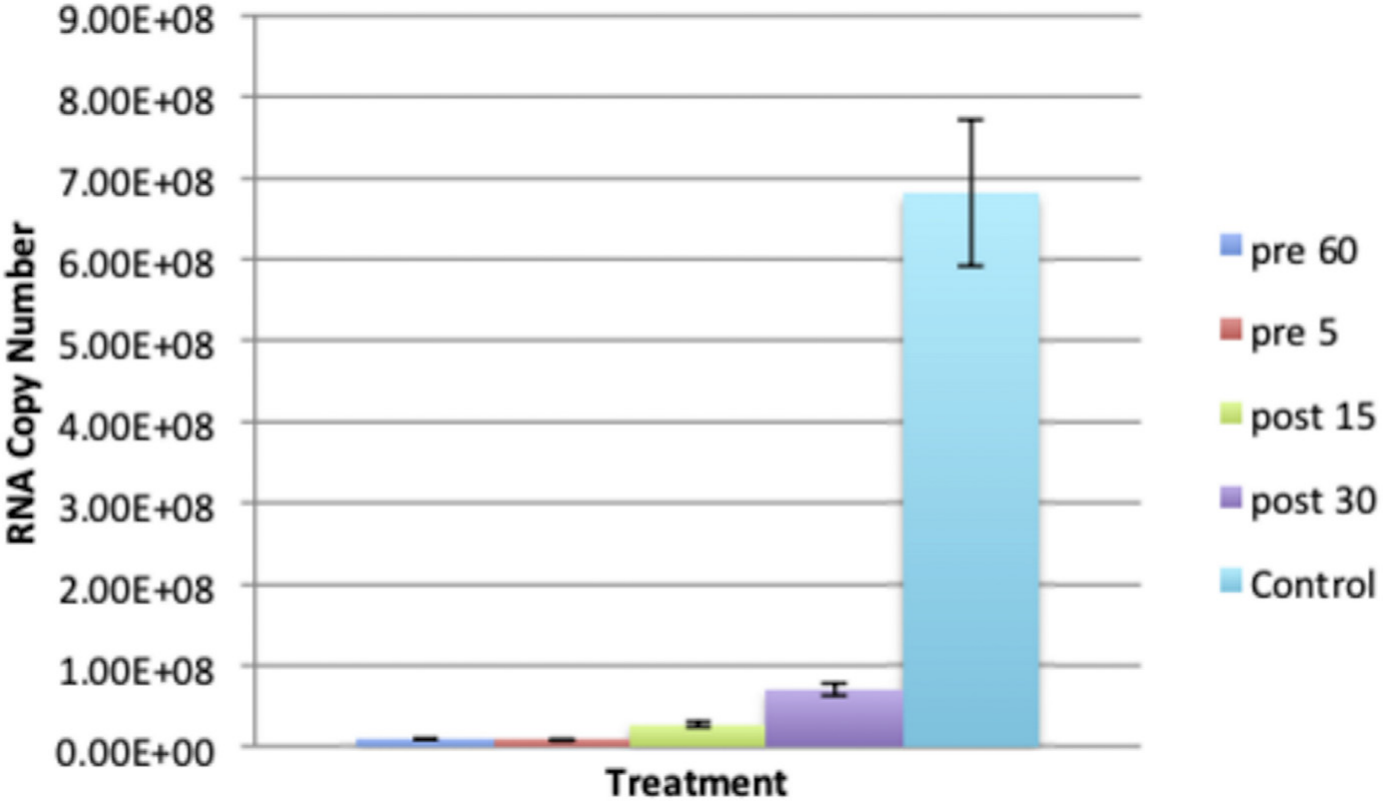
BAF time of addition assay. Identical BHK monolayers were treated with 100 nM BAF and infected with SVHR (MOI = 10 PFU/cell) at the indicated times and described orders. Infections took place and ambient temperature for 1 hour and then monolayers were transferred to 37 °C for 10 hours. After incubation, cells were processed for RT-qPCR as described. Results presented are an average of 3 biological replicates, error bars represent the standard error of these replicates.

Studies on the effect of BAF on alphavirus replication using radiolabeled RNA have been reported, but those experiments were only carried out to approximately 3.5–4 hours post infection (hpi) [9]. We monitored viral RNA replication of SVHR and BRSV in the presence and absence of BAF using RT-qPCR over the course of 10 hours. A concentration of 100 nM BAF and a treatment time of 1 hour were seen to be sufficient to nearly neutralize all acidic compartments. This was determined by incubating cell monolayers with acridine orange (5 μg/mL), which only labels acidic compartments [35]. No labeling was seen in BAF treated cells (Fig. 3) and this effect persisted past 24 hours after treatment (data not shown) [34]. In the absence of BAF treatment, assay of viral RNA synthesis of SVHR and BRSV infected cells showed that both virus infections approached a maximum RNA copy number of approximately 10^8^ molecules per approximately 9.5 × 10^5^ cells (Fig. 4). For SVHR, RNA production in the presence of BAF lagged behind production of SVHR RNA in the absence of drug by approximately 2 orders of magnitude at 3 hpi but slowly increased production over 10 hours. During the same time course, BRSV in the absence of drug produced amounts of RNA nearly equivalent to SVHR in the absence of drug. In the presence of BAF, on average BRSV produced approximately 1.7 × 10^4^, 1.2 × 10^6^, 4.1 × 10^6^, and 4.0 × 10^6^ copies of RNA per approximately 9.5 × 10^5^ cells at 3, 5, 7, and 10 hpi, respectively. These results indicate that previous assays conducted by others may have been prematurely terminated at a time point where a low level of RNA was produced; resulting in the conclusion that entry had not occurred. In this particular assay, it became difficult to measure RNA production beyond 10 hours because monolayers lost their integrity when cells lysis and detachment began, possibly skewing the measurement. To separate the apparent inhibition of RNA production from that of virus entry, a time of addition assay (Fig. 5) was conducted in which BAF was added to monolayers at time increments of 1 hour, 5 minutes prior to infection, or 15 minutes, and 30 minutes post infection. All infections were allowed to proceed to 10 hours before monolayers were processed for RT-qPCR. As anticipated, there was a significant reduction in RNA replication for the 1 hour pretreatment (reduction to approximately 1.5% of untreated control). RNA production was inhibited for the BAF treatment 5 minutes prior to infection (reduced to approximately 1.3% of untreated control), as well as the treatments at 15 minutes after infection (reduced to approximately 4% of untreated control) and 30 minutes after infection (reduced to approximately 10.5% of untreated control).

### 2.4 T15I/Y18H mutations are responsible for bypass in BAF-induced blockages of RNA synthesis

**Fig. 6:**
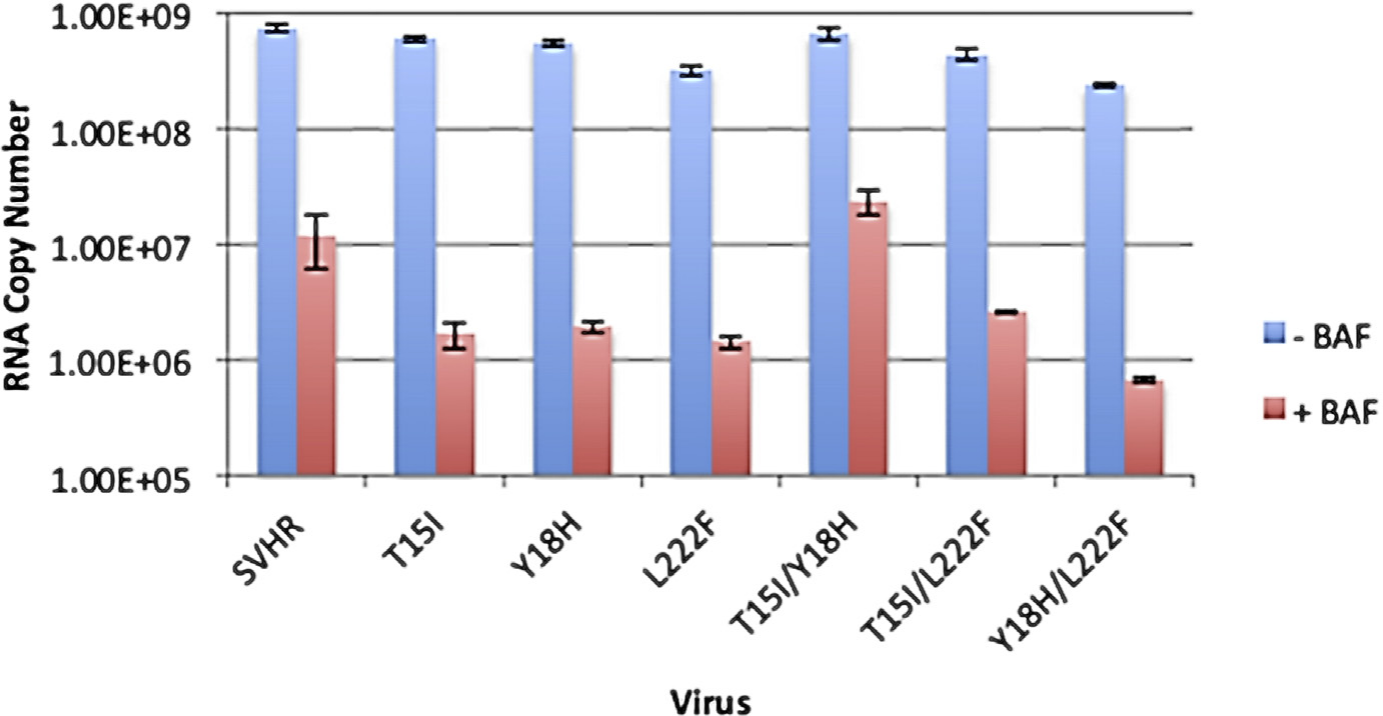
RNA production in BRSV-derived mutants. BHK cells were treated with BAF as above, infected with the indicated virus, and assayed for RNA production by RT-qPCR at 10 hpi. The control treatment group (no BAF) is shown in blue and cells treated with 100 nM BAF are shown in red. Data presented are an average of two biological replicates, error bars represent the standard error of these replicates.

To determine which of the three mutations make the greatest contribution to the BRSV phenotype we made single mutant strains of SVHR (E2:T15I, E2:Y18H, E1:L222F) and double mutant strains of SVHR (E2:T15I/Y18H, E2:T15I/E1:L222F, E2:Y18H/E1:L222F) that contain permutations of the single mutations. The mutants were then tested with regard to RNA synthesis (Fig. 6). Data are presented as a log_2_ transformation of the raw data. In the absence of BAF treatment, SVHR and all of the BRSV single- and double-mutants exhibited RNA production of similar amounts, about 2.9 × 10^1^ copies. (Fig. 6). However, with BAF treatment, E2:T15I had a reduction of RNA production to an average of 2.0 × 10^1^ copies, and E2:Y18H and E1:L222F had a decrease to 2.1 × 10^1^ and 2.0 × 10^1^ copies, respectively. Similarly, E2:T15I/E1:L222F and E2:Y18H/E1:L222F had a reduction of RNA production to 2.1 × 10^1^ and 1.9 × 10^1^ copies, respectively. By contrast, E2:T15I/Y18H only had RNA production reduced to 2.5 × 10^1^ copies. SVHR in the presence of BAF produced an average RNA copy number of 2.1 × 10^1^. The RNA levels of BAF-treated mutants examined in this experiment compare to the BAF-treated BRSV at 10 hpi. (Fig. 6). These mutants indicate that viral RNA encoding a threonine to an isoleucine at the 15^th^ position and a tyrosine to a histidine at the 18^th^ position of the E2 gene is sufficient to confer the partial ability to bypass BAF-induced blockages of RNA replication.

### 2.5 T15I/Y18H mutations are responsible for the ability to bypass blockages of BAF in virus egress

**Fig. 7:**
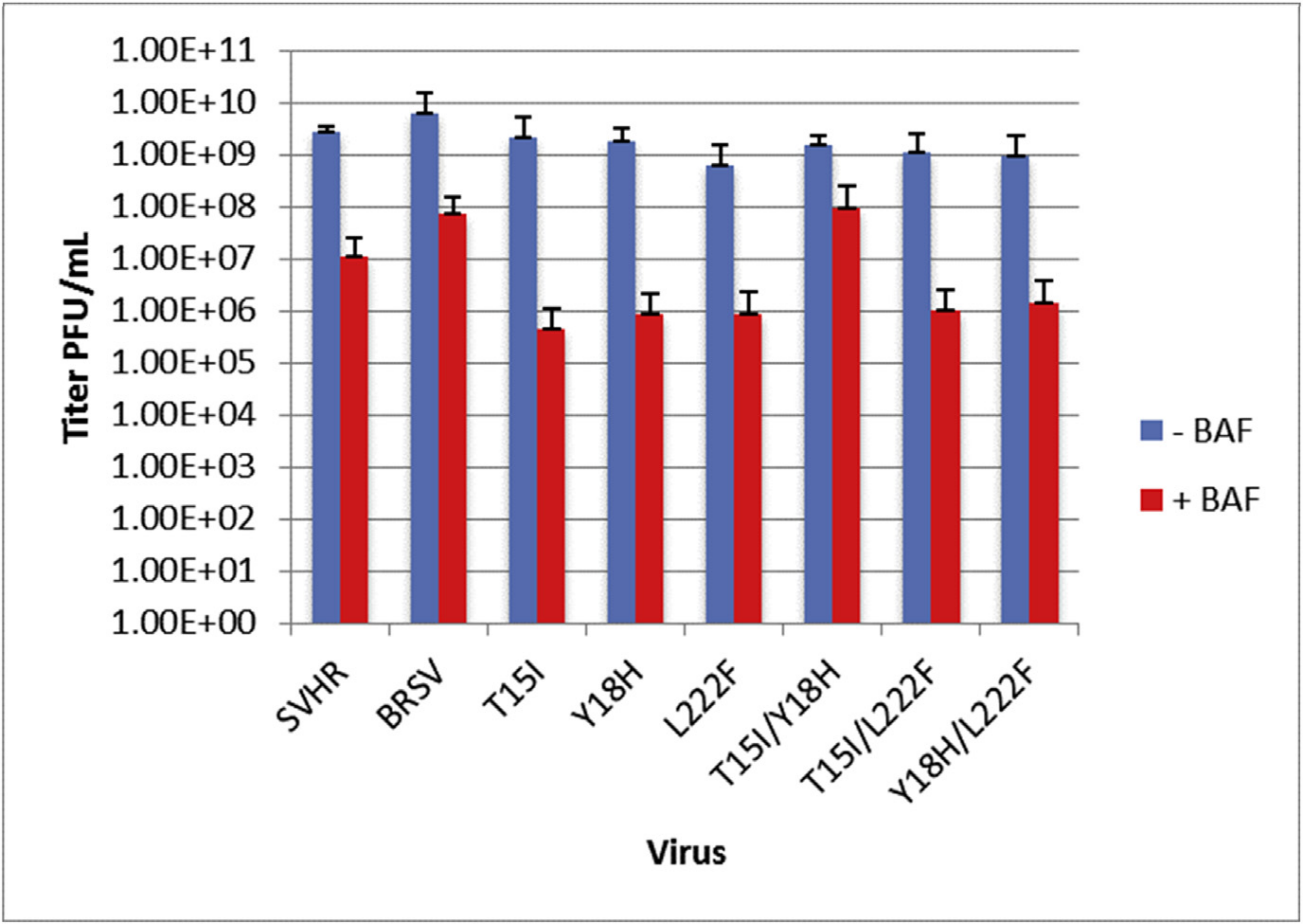
Mature virus production from BRSV-derived mutants. BHK cells were treated with 100 nM BAF for 1 hour. at room temperature, infected at an MOI of 10, and assayed for mature virus production by titration of the 20 hpi sample. The control treatment group (no BAF) is shown in blue and cells treated with 100 nM BAF are shown in red. Error bars represent the standard error of three biological replicates.

To determine which mutations have the greatest contribution to the BRSV phenotype with regard to virus egress (Fig. 7), the single mutant strains of SVHR (E2:T15I, E2:Y18H, E1:L222F) and double mutant strains of SVHR (E2:T15I/E2:Y18H, E2:T15I/E1:L222F, E2:Y18H/E1:L222F) that contain permutations of the single mutations described above were again used (those presented in Fig. 6). Data are presented as a log_2_ of the raw data. In the absence of BAF, all viruses produced mature virus with an average titer of ∼3 × 10^1^ PFU/mL. BAF treatment caused a dramatic reduction in titer to approximately 1.7–1.8 × 10^1^ PFU/mL for E2:T15I, E2:Y18H, E1:L222F, E2:T15I/E1:L222F, and E2:Y18H/E1:L222F. The E2:T15I/E2:Y18H mutant produced a titer that was approximately 2.3 × 10^1^ PFU/mL in the presence of drug. BRSV, in the presence of BAF produced an average titer of approximately 2.6 × 10^1^ PFU/mL (Fig. 7). This result indicates that the E2:T15I/E2:Y18H mutations confer the partial ability to bypass BAF-induced blockages in viral egress.

### 2.6 Effect of BAF on virus assembly and egress

**Fig. 8:**
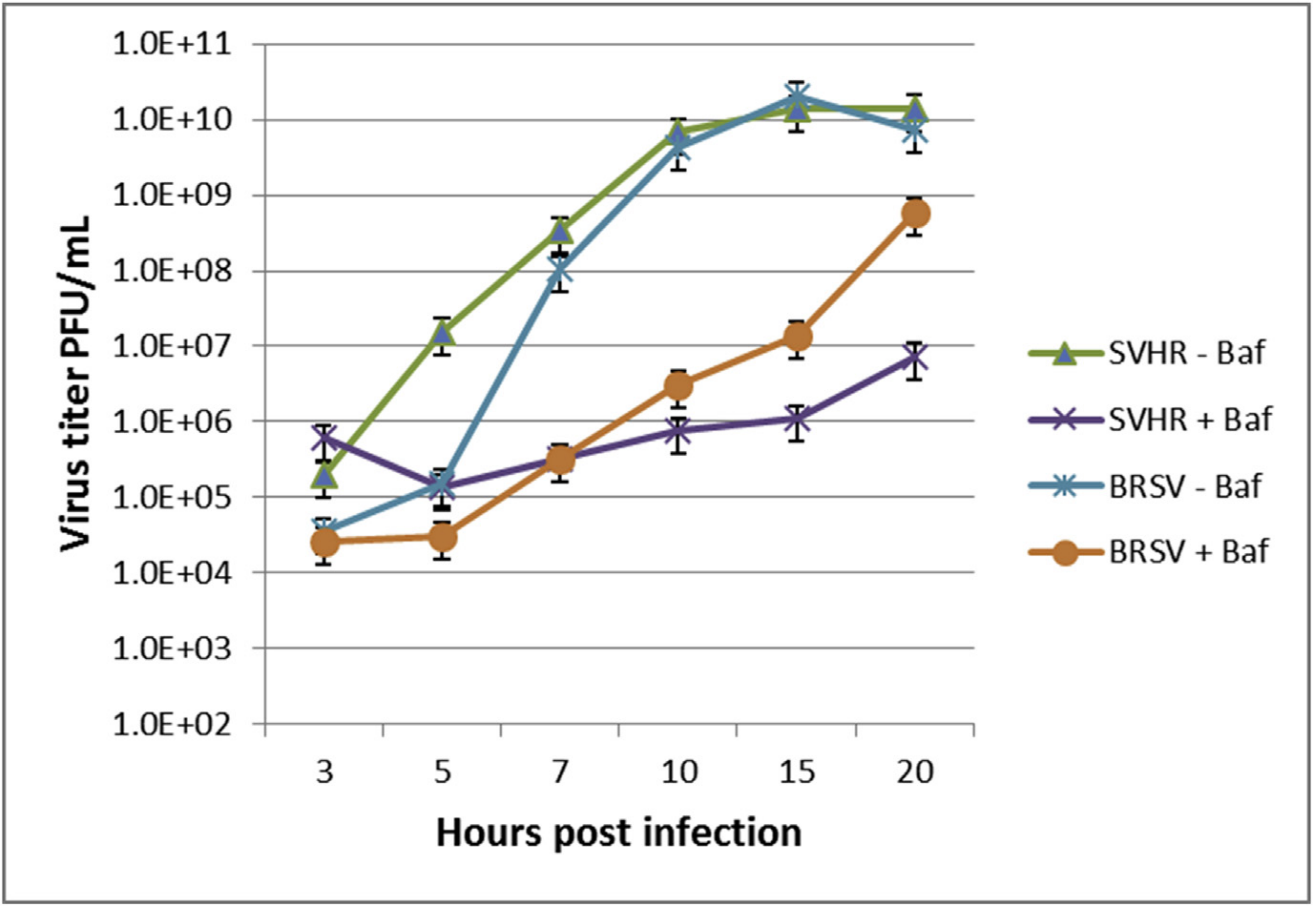
The effect of BAF on mature virus production of SVHR or BRSV. BHK cells were either untreated or treated with 100 nm BAF at room temperature for 1 hour. Monolayers were then infected in presence or absence of BAF for 1 hour. Monolayers were incubated at 37 °C for the indicated amount of time and the resulting supernatant was then titered. Treatment groups are as follows: blue, SVHR without BAF; red, SVHR + 100 nM BAF; green, BRSV without BAF; purple, BRSV + 100 nM BAF. Experiment is representative of two biological replicates.

To characterize what effects an inhibited V-ATPase had on egress of the viruses in question, we determined the titer of the progeny viruses on BHK monolayers after SVHR or BRSV was released from control or BAF-treated cells during a 20 hr. time course (Fig. 8). The titers of BRSV under both treatments and BAF treated-SVHR were all within about one order of magnitude from the SVHR control at 3 hpi. Greater disparity between the treatment groups began around 5 hpi. BRSV in the absence of BAF at 20 hpi was produced at titers comparable to the untreated SVHR control > 10^10^ PFU/mL. With BAF treatment, both SVHR and BRSV had a reduction of approximately 4 and 2 orders of magnitude, respectively, by 20 hpi compared to the untreated groups. The titers of virus produced at the end of 20 h were 4x10^6^ PFU/mL for SVHR and 6x10^8^ PFU/mL for BRSV in the presence of drug. We confirmed that at the 10 hpi time point BAF treatment did not produce excessive empty virus particles in any of the treatment groups by comparing the RNA levels to the virus titer in PFU/mL (data not shown). Thus, BAF treatments did not contribute to an altered particle to PFU ratio. The genome-to-PFU ratios calculated were approximately between 10 and 20 for all treatment groups.

**Fig. 9:**
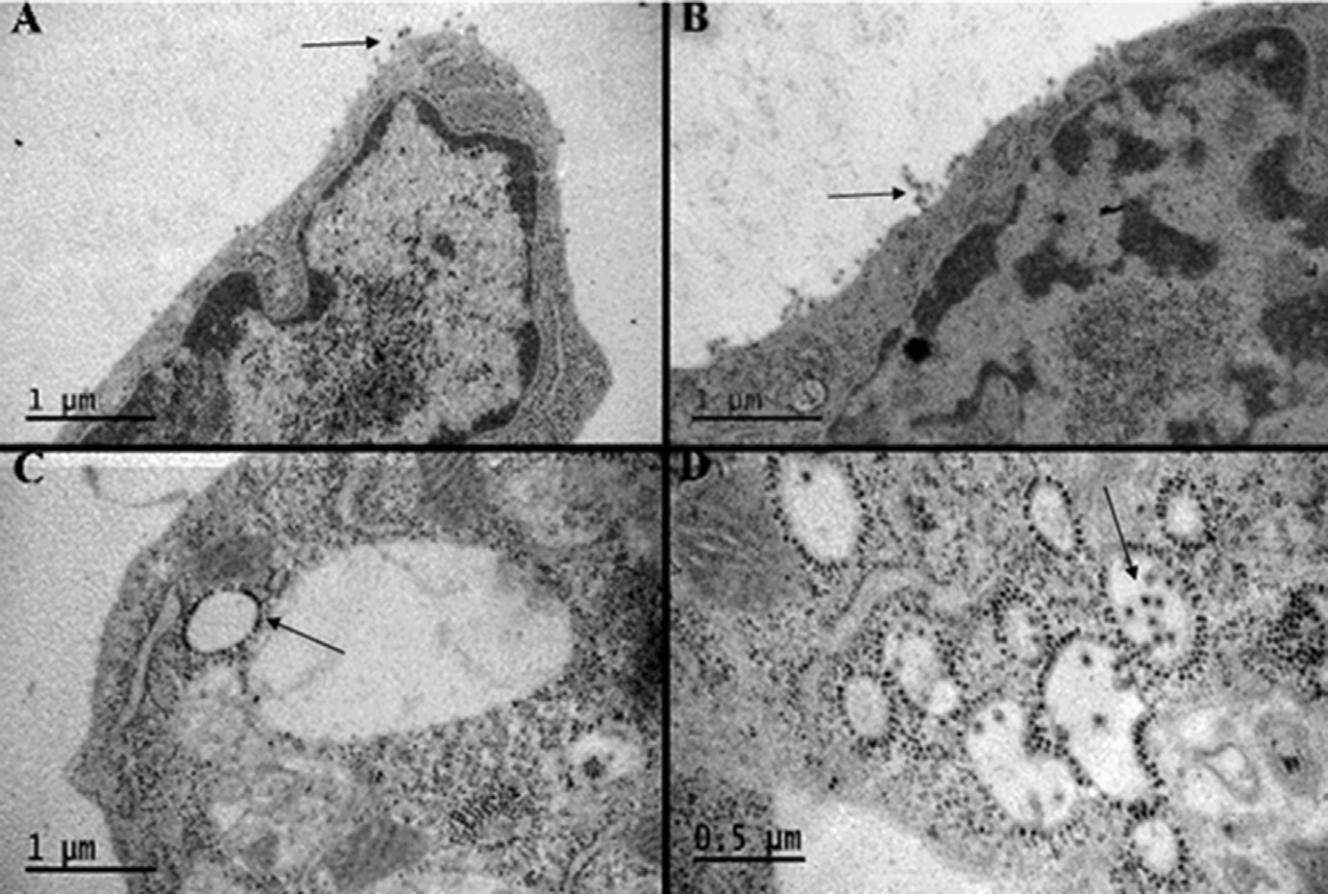
The effect of BAF on viral assembly of SVHR or BRSV. Thin section electron micrographs of BHK cells infected with SVHR in the absence of BAF (A), BHK cells infected with BRSV in the absence of BAF (B), BHK cells infected with SVHR in the presence of 100 nM BAF (C), BHK cells infected with BRSV in the presence of 100 nM BAF. The arrows in A and B point to mature virus budding from the surface of cells. The arrow in C points to nucleocapsids bound to the surface of vesicular membranes. The arrow in D points to mature virus budding into the *lumen* of a vesicle. All infections were done at an MOI of 10 at room temperature for 1 hour. After infection, monolayers were moved to 37 °C for approximately 18 hours. After incubation, monolayers were processed for electron microscopy as described. This experiment was performed at least 3 times and the images provided are representative observations.

To determine the ultimate fate of maturing virus particles, observations of monolayers 20 hpi, of SVHR and BRSV under the same experimental conditions as described for the egress experiment, were made using electron microscopy (Fig. 9). BRSV and SVHR are seen to bud normally from the cell surface in the absence of BAF (Fig. 9 A, B). However, SVHR and BRSV nucleocapsids accumulate at the surface of vesicular bodies when infected in the presence of BAF (Fig. 9 C, D). In BAF-treated cells that have been infected with BRSV, the virus is able to overcome the block in maturation and is enveloped in the membrane of the internal vesicles. Release of mature virions occurs as cells succumb to the rigors of infection, drug treatment, and lysis.

## 3 Discussion

The goal of this study was to characterize the involvement of V-ATPase in the replication cycle of Sindbis virus. This was accomplished by observing the effects of various inhibitors on virus entry using electron microscopy and by producing a BAF-resistant mutant virus. The phenotypes of the two viruses were determined in the context of RNA replication and mature virus production in the presence and absence of BAF. Early and late stages in the viral replication cycle were monitored using RT-qPCR, virus titration by plaque assay, and electron microscopy.

Our selection of metabolic inhibitors for the EM studies (Fig. 2) is represented by an inhibitor of endocytosis (cytochalasin-D) [36], a eukaryotic translation inhibitor (cyclohexamide) [37], a caveolae-mediated endocytosis inhibitor (genistein) [38], two ionophores (CCCP and monensin) [39, 40], and a V-ATPase inhibitor (BAF) [28]. The observation that empty virus particles were produced found bound to the cell plasma membrane using our EM protocol after treatment with cytochalasin-D is direct evidence that the *Alphavirus* RNA can enter the cells by pathways not requiring receptor-mediated endocytosis. These observations confirm our previous observations that virus entry can occur at the cell surface [5] and that the V-ATPase has some effect on the replication process independent of endosome pH or virus penetration. As proposed by others, V-ATPase could participate in other aspects of the virus replication cycle. For instance, V-ATPase has been shown to reduce egress of Japanese encephalitis virus [41], dengue virus [42], vesicular stomatitis virus [41], and herpes simplex virus [41].

One mechanism by which viral RNA could be introduced into a host cell is by physical translocation. It was considered possible that an enzymatic component of the cell, like a ribosome in close proximity to the cytoplasmic side of the plasma membrane, could attach to viral RNA and facilitate its entry into the cell in an event concurrent with viral protein translation. This event would be mechanistically similar to the transcription-dependent entry event of bacteriophage T7 [43, 44, 45]. This does not seem to be the case because cyclohexamide treatment, which inhibits protein synthesis, only reduced the number of empty virus particles by 20% during the 15-minute infection period (Fig. 2) while reducing virus production ten-fold [5].

The observation that RNA entry follows Arrhenius kinetics with a low activation energy of 27 Kcal/mol suggests that there is an enzymatic component involved with genome entry and that genome entry is not physically forced, as is the case with T4 bacteriophage [46, 47]. The treatment of the monolayers with ionophores shows a more dramatic reduction in virus entry (a reduction of approximately 40% for CCCP and monensin) compared to the other treatments. Taken together with the observation that the kinetics of entry follows Arrhenius kinetics, and is inhibited by ionophores, these data suggest the entry event relies partly on an unidentified protein component and a gradient of ions across the cell membrane, an observation that has been made previously [48].

The generation of a BAF resistant mutant (BRSV) raises interesting questions to consider for the mechanism of infection and replication of the *Alphaviruses*. Certainly, there are countless studies that investigate the roles of membrane fusion and endosomal pH in the receptor mediated endocytosis of Sindbis virus [9, 49, 50, 51, 52, 53, 54, 55, 56, 57]; these observations do not preclude the possibility that the virus *can* enter through other routes, such as direct penetration [2, 5, 6, 34, 58]. Previous studies have set out to characterize the intra- and intermolecular architecture of the *Alphavirus* structural proteins and because of these, questions have arisen regarding the functional role of the specific mutations found in BRSV [3, 59, 60, 61]. It has been suggested, because of these previous studies, that the mutations that have arisen in the analogous BRSV mutant, SFV fus-1, merely act to destabilize the interactions of the fusion proteins, thereby increasing the pH at which fusion occurs [3, 59, 60, 61]. This situation can certainly be true in the context of the receptor-mediated endocytosis model, but considering the direct, morphological evidence provided by this study, and studies referenced in this work, we believe these criticisms to be peripheral and are not evidence that direct penetration at the plasma membrane is precluded. In addition, our immunofluorescence experiment (Fig. 3) with SVHR and BRSV shows that BRSV mutations *do not* cause fusion to occur at neutrality. If this were the case, one would expect the entire BAF-treated monolayer to be infected with BRSV. This is supported by the fact that alphavirus infection has been shown to be a process that eventually allows for ions to leak through the PM [62, 63, 64].

It is also important to point out that selection of an SFV mutant requiring lower than normal pH for fusion also produced the mutant T15I. The studies that attempted to characterize the intermolecular contacts between the proteins of glycoprotein spikes involve the fitting of recombinant-expressed fused ectodomains of the envelope proteins that are then fitted into densities acquired from cryo-electron microscopy reconstructions of whole virus particles [3]. Structural studies such as these may reveal sound information about intramolecular contacts; but because of the lack of the viral membrane, the native super-structure of the glycoprotein coat may be compromised. Furthermore, Whitehurst, *et al.* have shown that a disulfide bridge proposed by the crystal structure of the E1 protein does not exist in the intact virion [65]. For these reasons, it may be possible that the BRSV mutations are not involved in protein-protein interactions within the glycoprotein spike and therefore do not destabilize the interactions with E1.

The phenotype of BRSV supports the hypothesis that the *Alphaviruses* can enter cells by direct penetration because infection took place in the *absence* of endosome acidification but with reduced levels of RNA replication [5]. A nonfunctional V-ATPase did not inhibit the synthesis of viral RNA for BRSV, but suppressed RNA synthesis for SVHR (Fig. 4). Similarly, a pretreatment of monolayers with BAF suppresses chikungunya virus RNA replication after entry occurs (data not presented). A functional V-ATPase also appears to be involved in viral maturation and egress. After determining the amount of mature virus produced in the presence and absence of BAF, we observed there was a large difference (∼2.5 orders of magnitude) in the amount of mature virus that was released from the SVHR and BRSV-infected cells (Fig. 8). The fact that RNA replication was inhibited by BAF addition *after* the monolayers were inoculated with virus shows that the suppression of RNA synthesis seen in this study is not due to an interruption in virus entry (Fig. 5). It has been demonstrated by others that at least half of all virus has entered the cell by 10–15 minutes after addition of virus to cell monolayers and entry has reached near completion by 30 minutes [66]. Studies involving direct observation by electron microscopy revealed that 50% of attached particles released their RNA in 15 min. at room temperature [5]. Additionally, reduction in RNA replication with the presence of BAF only 5 minutes before infection, a time where pH neutralization has not taken place (assayed by acridine orange, data not shown), indicates that a functional ATPase, not low pH, is required for the efficient initiation of RNA synthesis.

We turned to electron microscopy to determine any differences in viral maturation because of the relatively large amounts of BRSV RNA produced together with a deficit of mature virus particles (Figs. 8 and 9). The budding of BRSV virus particles into the *lumen* of the vesicles of BAF-treated cells demonstrates that these mutations allow the virus to bypass the normal maturational pathway. The virus is likely released as the cells break up during an extended incubation period. The BRSV mutations confer a property to the virus such that when the primary pathway of maturation—specifically, the secretory pathway of the cell—is inhibited, a secondary pathway can be utilized. In agreement with prior studies [41, 42, 67], this inhibition of the secretory pathway by BAF accounts for the lag in Sindbis virus production, seen in Fig. 8. It appears that both of the mutations in E2 (T15I/Y18H) provide a significant contribution to the ability of the virus to resist the RNA-suppressing consequence of an inhibited V-ATPase as well as the maturational obstructions (Figs. 6 and 7). The E2:T15I mutant seems to have a relatively high efficiency of virus maturation compared to the other mutants, with the exception of the E2:T15I/E2:Y18H double mutant.

**Fig. 10:**
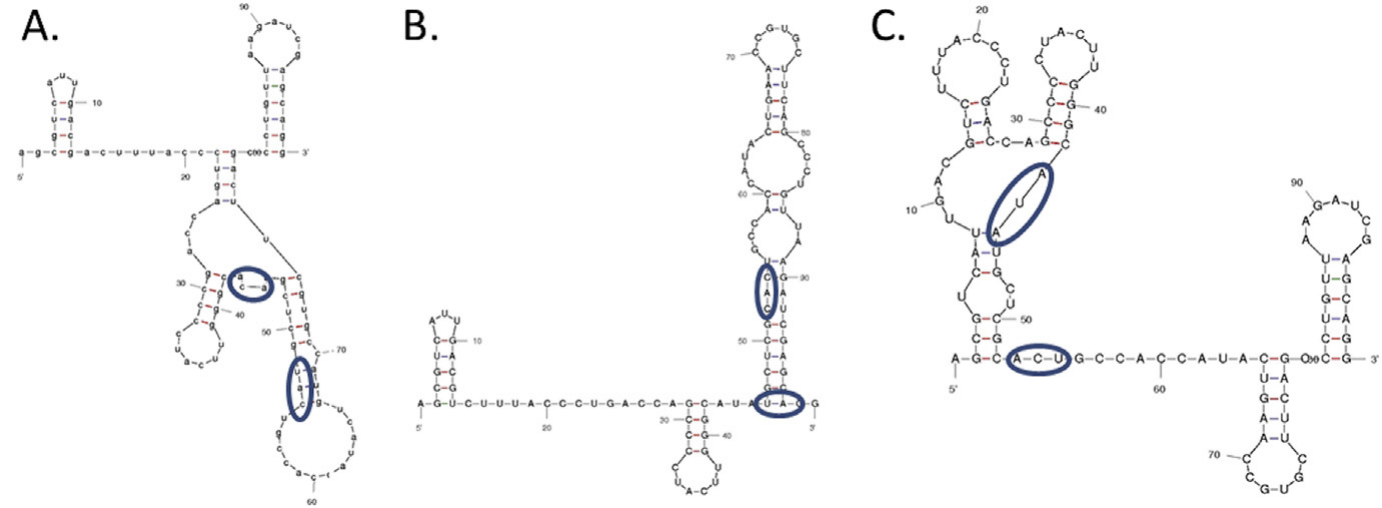
Optimum RNA folds at 37 °C for SVHR ΔG = −21.18 (A), BRSV ΔG = −23.8 (B), and BRSV ΔG = −20.38 (C). These models of the E2 protein encompassing amino acid positions 15 and 18 suggest that the T15I and Y18H mutations may alter the secondary structure of this region of the RNA. These predicted conformational changes may recruit a different complement of host cell proteins that allow for efficient replication in the absence of an active V-ATPase. Fold models were made using Mfold [71]. The blue circles indicate the sequences changed in the BRSV mutant.

**Fig. 11:**
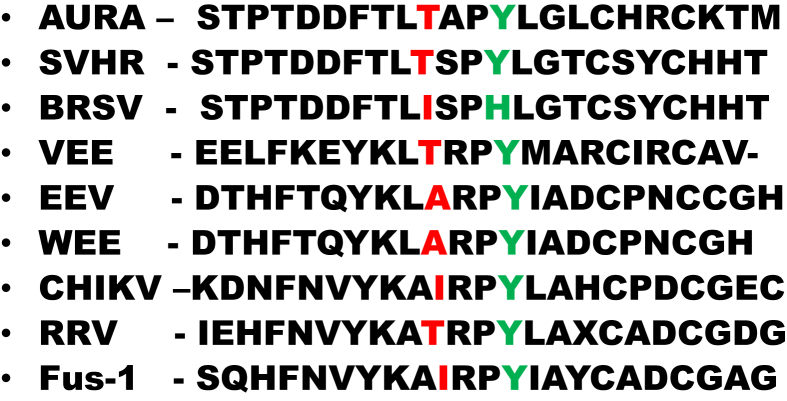
Alignment of selected alphaviruses. This alignment of approximately 20–25 amino acids shows the region of the E2 protein containing the T15Y and Y18H mutations of BRSV (shown by arrows) in comparison with other alphaviruses. The tyrosine at position 18 is strictly conserved amongst the alphaviruses. Sequence of BRSV and SVHR are from this research. The other sequences are from reference [61].

Because the E2:T15I/E2:Y18H mutant retains a total RNA producing activity greater than the other mutants and because both of these mutations lie in the E2 structural protein, we suggest that a change in secondary structure in the E2 gene RNA sequence may provide a *cis*-acting element that facilitates RNA synthesis in the altered cytoplasmic environment (Fig. 10) [68]. Additionally, the altered sequence of E2 protein, once translated, may similarly recruit additional or different host factors involved in viral maturation in the absence of a functional V-ATPase. A previously published article provides evidence that the V-ATPase is involved in the egress of dengue virus [42]. In our experiments, SVHR entry has clearly occurred in the presence of BAF, but the BRSV mutations are required for a productive infection. Finally, serially passaging BRSV in the absence of BAF did not result in the restoration of the SVHR sequence (data not shown). This indicates that the mutations in BRSV have no negative effect on virus replication in the absence of drug. The single mutant E2:T15I is particularly interesting. A previous study in which Semliki Forest virus was mutated (fus-1) to require a lower than normal pH in order to fuse artificial liposomes also resulted in this same amino acid change which aligns to the same position in the alphavirus genome (Fig. 11). It was also shown that fus-1 infection was more sensitive to NH_4_Cl concentration, indicating a more acidic environment was required for infection [61]. In our hands, this single mutant grew in the presence of BAF, indicating that it is infecting cells at a higher pH than would be expected in light of the fus-1 study. This observation, in addition to others [69], separates the events surrounding virus entry into host cells from those related to *in vitro* fusion of virus with liposomes [70]. Both the fusogenic mutant, designated fus-1, and our BAF-resistant mutant have a threonine-to-isoleucine mutation at a conserved residue, E2-15. The fus-1 mutant was shown to revert back to the wild type sequence during infections in the presence of ammonium chloride. Our findings indicate that there is no selective pressure to favor the SVHR wild type sequence in the absence of BAF and suggests that the mechanism of action of ammonium chloride inhibition is quite different than that of BAF. Finally, the mutations E2:T15I/Y18H give no apparent selective advantage to the virus except in the unnatural situation of a nonfunctional V-ATPase. Published sequences of *Alphaviruses* indicate that the combination of amino acid changes in BRSV does not exist in examined variants of natural virus populations.

## 4 Methods

### 4.1 Tissue culture

BHK-21 cells were cultured in minimal essential medium (MEM) supplemented with 10% fetal bovine serum, 5% tryptose phosphate broth, 2 mM glutamine, 50 μg/mL gentamicin, and 10 mM HEPES (pH 7.4). Cells were maintained at 37 °C with 5% CO_2_.

### 4.2 Drug treatment, infections, acridine orange staining, and electron microscopy

BHK cells were cultured in 6-well plates until ∼90% confluent. For infections in the presence of BAF, monolayers were incubated with complete MEM containing 100 nM BAF for 1 hour at RT. Virus was diluted in MEM in the presence or absence of 100 nM BAF to give a final MOI of approximately 10 for BRSV and SVHR. Cells were infected for 1 hour at RT (25 °C). Inoculum was removed after infection and replaced with MEM either containing or lacking 100 nM BAF (Tocris). Monolayers were incubated for indicated times at 37 °C. Live cell monolayers were stained with acridine orange (Fig. 3) as described previously to verify the activity of BAF [35]. BHK cell monolayers were processed as described previously [5].

### 4.3 Immunofluorescence assay

BHK monolayers were infected as above. After 10 hours, the monolayers were fixed with 100% ice-cold methanol. The monolayers were then incubated with anti-SVHR (1:1000) in PBS supplemented with 0.1% BSA for 1 hour at ambient temperature. The monolayers were then carefully washed with PBS supplemented with 0.1% BSA three times. The monolayers were then incubated with and Alexafluor-conjugated secondary antibody for one hour and then the monolayers were washed as before. The monolayers were then left in PBS for observation under UV light.

### 4.4 RT-qPCR

All samples for RT-qPCR were extracted from monolayers of 6-well plates after appropriate treatment. Extraction was accomplished with Trizol (Thermo-Fisher) using manufacturer's instructions. Following extraction, RNA concentrations were normalized to total RNA concentration. cDNA of each sample was made using random hexamer primers from the Maxima First Stand cDNA Synthesis Kit (Thermo) following the instructions of the manufacturer. cDNA was then either stored at −80 °C or used for the Real Time PCR. RT-qPCR on all samples was done on an AB 7500 Fast Dx instrument (Applied Biosystems) with the following primers and TaqMan probe:

SVHR Sense: 5′-TGTGTACACCATCTTAGC-3′
SVHR Antisense: 5′-CAAAGGTATGCACAACTG-3′
SVHR Probe: 5′-FAM-AGTGCCTGACGCCATA-MGB-NFQ-3′

Samples were prepared in TaqMan Fast Advanced Mastermix (Thermo) following the instructions of the manufacturer and the following 40-round cycle was used for amplification: 95 °C, 3 sec; 60 °C, 30 sec. Results were analyzed using the 7500 Fast System v1.4.0 software (Applied Biosystems). All RNA replication experiments were analyzed against a standard curve made from the purified Toto1101 infectious clone for SVHR.

### 4.5 Titration by plaque assay

Viral titrations were done on BHK cells in 6-well plates that were ∼95% confluent. 250 μL of virus from each 10-fold serial dilution was added to the monolayers and allowed to adsorb for 1 hour at RT. Following infection, the supernatant was replaced with 2 mL of 1X MEM containing 1% agarose. The assays were allowed to incubate for approximately 18 h at 37 °C. Following this incubation, a 2 mL overlay of 1X PBS-D, 1% agarose, and 0.06% phenol red was added to the wells and allowed to incubate for 4 hour at 37 °C protected from light. Visible plaques were subsequently counted.

### 4.6 Entry assay

Cells were treated with the indicated drug for 1 hour. The monolayers were infected for 15 min at an MOI of 1000 PFU/cell at room temperature, and processed as described [5, 6].

### 4.7 Generation of BRSV mutant and site-directed mutagenesis

Twenty-two serial passages of SVHR in BHK cells treated with 100 nM BAF produced a mutant that was resistant to BAF. The BHK cells were pretreated with BAF for 1 hour at room temperature and then infected with SVHR. After the 1 hour viral adsorption at room temperature, the flask was transferred to 37 **°**C for ∼24 hours after which the supernatant was harvested. BHK cells were infected with BRSV and the RNA extracted using Trizol (see RT-qPCR above). The resulting RNA was deep sequenced and the resulting contigs. compared to the Toto1101 sequence (accession J02363). A build of the resulting sequence data in CLC workbench compared to Sindbis virus strains in the database generated a table of variants differing from the reference strain. Only sequence data which was not from a Sindbis variant or resulted in the conservation of the known amino acid sequence was considered a mutation. Three mutations in the structural proteins were thus identified: one in E1 (L222F) and two in E2 (T15I, Y18H). There were no mutations found in the replication (nonstructural) proteins. These mutations were further studied as single and multiple virus templates.

Construction of the BRSV single and double mutants was accomplished with PfuTurbo (Agilent Technologies) using the manufacturer's instructions and the following primers on a Toto1101 template:

E2_T15I_FWD: 5′-CCCCTACTTGGGCATATGCTCGTACTGCC-3′
E2_T15I_REV: 5′- GGCAGTACGAGCATATGCCCAAGTAGGGG-3′
E2_Y18H_FWD: 5′- TTGGGCACATGCTCGCACTGCCACCATACTG-3′
E2_Y18H_REV 5′- CAGTATGGTGGCAGTGCGAGCATGTGCCCAA-3′
E2_T15I/Y18H_FWD: 5′- GCCCCTACTTGGGCATATGCTCGCACTGCCACCAT-3′
E2_T15I/Y18H_REV: 5′-ATGGTGGCAGTGCGAGCATATGCCCAAGTAGGGGC-3′
E1_L222F_FWD: 5′- AGCACAGACATTAGGCTATTCAAGCCTTCCGCC-3′
E1_L222F_REV: 5′- GGCGGAAGGCTTGAATAGCCTAATGTCTGTGCT-3′

## Declarations

### Author contribution statement

Ryan M. Schuchman: Conceived and designed the experiments; Performed the experiments; Wrote the paper.

Ricardo Vancini, Amanda Piper, Denitra Breuer, Mariana Ribeiro, Joseph Magliocca, Veronica Emmerich: Performed the experiments.

Davis Ferreira: Conceived and designed the experiments; Performed the experiments; Analyzed and interpreted the data.

Raquel Hernandez, Dennis T. Brown: Conceived and designed the experiments; Analyzed and interpreted the data; Wrote the paper.

### Funding statement

This work was supported by the Clayton Foundation for Research, Carson City, NV; and by the North Carolina Agriculture Research Service.

### Competing interest statement

The Authors declare no conflict of interest.

### Additional information

No additional information is available for this paper.
